# Comparison of Imported *Plasmodium ovale curtisi* and *P. ovale wallikeri* Infections among Patients in Spain, 2005–2011

**DOI:** 10.3201/eid2003.130745

**Published:** 2014-03

**Authors:** Gerardo Rojo-Marcos, José Miguel Rubio-Muñoz, Germán Ramírez-Olivencia, Silvia García-Bujalance, Rosa Elcuaz-Romano, Marta Díaz-Menéndez, María Calderón, Isabel García-Bermejo, José Manuel Ruiz-Giardín, Francisco Jesús Merino-Fernández, Diego Torrús-Tendero, Alberto Delgado-Iribarren, Mónica Ribell-Bachs, Juan Arévalo-Serrano, Juan Cuadros-González

**Affiliations:** Príncipe de Asturias University Hospital, Madrid, Spain (G. Rojo-Marcos, J. Arévalo-Serrano, J. Cuadros-González,);; Instituto de Salud Carlos III, Madrid (J.M. Rubio-Muñoz); Carlos III Hospital, Madrid (G. Ramírez-Olivencia);; La Paz University Hospital, Madrid (S García-Bujalance);; Doctor Negrín University Hospital, Las Palmas de Gran Canaria, Spain (R. Elcuaz-Romano);; Ramón y Cajal Hospital, Madrid (M. Díaz-Menéndez);; Gregorio Marañón University Hospital, Madrid (M. Calderón);; Getafe University Hospital, Madrid (I. García-Bermejo);; University Hospital of Fuenlabrada, Madrid (J. M. Ruiz-Giardín);; Severo Ochoa University Hospital, Madrid (F.J. Merino-Fernández);; University General Hospital of Alicante, Alicante, Spain (D. Torrús-Tendero);; University Hospital Fundación Alcorcón, Madrid (A. Delgado-Iribarren);; Hospital General de Granollers, Barcelona, Spain (M. Ribell-Bachs).

**Keywords:** Plasmodium ovale curtisi, Plasmodium ovale wallikeri, imported malaria, epidemiology, clinical features, Spain, parasites, vector-borne infections, malaria, thrombocytopenia

## Abstract

Sequencing data from *Plasmodium ovale* genotypes co-circulating in multiple countries support the hypothesis that *P. ovale curtisi* and *P. ovale wallikeri* are 2 separate species. We conducted a multicenter, retrospective, comparative study in Spain of 21 patients who had imported *P. ovale curtisi* infections and 14 who had imported *P. ovale wallikeri* infections confirmed by PCR and gene sequencing during June 2005–December 2011. The only significant finding was more severe thrombocytopenia among patients with *P. ovale wallikeri* infection than among those with *P. ovale curtisi* infection (p = 0.031). However, we also found nonsignificant trends showing that patients with *P. ovale wallikeri* infection had shorter time from arrival in Spain to onset of symptoms, lower level of albumin, higher median maximum core temperature, and more markers of hemolysis than did those with *P. ovale curtisi* infection. Larger, prospective studies are needed to confirm these findings.

Malaria caused by *Plasmodium ovale* infection has been considered a low-prevalence disease with limited geographic distribution, benign clinical course, and easy treatment; therefore, little attention has been paid to it. Diagnosis of *P. ovale* malaria can be difficult because of low parasitemia levels, mixed infections with other *Plasmodium* species, and false negatives from malaria rapid diagnostic tests (RDTs) (*1*). However, recent epidemiologic studies conducted by using PCR techniques have found *P. ovale* infections in most of sub-Saharan Africa, Southeast Asia, and the Indian subcontinent (*2*–*5*), including prevalence as high as 15% according to results of cross-sectional studies conducted in rural Nigeria (*6*) and Papua New Guinea (*7*). In addition, severe complications such as spleen rupture, severe anemia, or acute respiratory distress syndrome (ADRS) (*8*) may occur in patients with *P. ovale* malaria. Thus, the global burden of *P. ovale* infection might have been underestimated.

On the basis of differences in its gene sequences, *P. ovale* was considered to be dimorphic or to comprise 2 subspecies (*2*,*3*,*9*,*10*). This difference has hampered molecular diagnosis in some cases because of lack of DNA amplification by PCR with gene-specific primers for the small subunit ribosomal RNA (ssrRNA) (*10*). These subspecies had been named classic and variant *P. ovale*, but a comprehensive study recently described differences between these subspecies in at least 6 genes (*4*). These findings demonstrate that *P. ovale* actually consists of 2 subspecies that co-circulate in Africa and Asia and that are unable to recombine genetically; the differences seem to be explained by real biological factors, rather than ecologic or geographic factors (*11*). *P. ovale curtisi* and *P. ovale wallikeri* were the names proposed for these species (*4*). 

Scant information is available on differences in clinical and analytical features, relapse profile, or accuracy of RDT results between these proposed species. Relatively high parasitemia levels were found in some patients with *P. ovale wallikeri* infection in Thailand (*12*), Vietnam (*13*), and Flores Island (*14*). A study published from a disease-endemic area of Bangladesh reported on the clinical features and degree of parasitemia in 13 patients with *P. ovale wallikeri* infection and 10 with *P. ovale curtisi* infection (*5*). These infections were diagnosed by PCR; only 4 of the 23 patients were symptomatic. Another recent study compared parasitemia levels, RDT results, and patient country of origin for 31 patients from Côte d’Ivoire and the Comoros Islands with imported *P. ovale wallikeri* infection and 59 with *P. ovale curtisi* infection, but no clinical data were provided (*15*). Clearly, information on these infections is limited.

Growth in international travel and migration has increased the incidence of imported malaria in industrialized countries. *P. ovale* infection may represent up to 8% of imported malaria cases, according to some published series of patients primarily from West Africa (*16*,*17*), where the proportion of sub-Saharan immigrants is high and PCR has been systematically performed. Yet, it is difficult to gather a substantial number of cases with clinico-epidemiologic correlation and molecular data. To identify clinical or analytical differences between *P. ovale wallikeri* and *P. ovale curtisi* infections and expand data on these infections, we conducted a multicenter, retrospective, comparative study of imported *P. ovale* infections diagnosed in Spain during 2005–2011.

## Methods

### Sample Selection

During June 2005–December 2011, blood samples from all patients with positive PCR results for imported infection with *P. ovale* were sent from public hospitals in Spain to the reference Malaria & Emerging Parasitic Diseases Laboratory of the National Centre of Microbiology in Madrid. The samples were shipped to the laboratory 1) to confirm the diagnosis of malaria and the species or 2) to study fever, anemia, or suspected malaria in patients with negative results on thick and/or thin smears and RDTs who were considered at high risk for malaria (i.e., immigrants and travelers to malaria-endemic areas).

### Microbiological Diagnosis

The initial diagnosis of imported *Plasmodium* spp. infection was made by thick and/or thin smears and/or by using the second-generation RDT NOW Malaria Test Kit (Binax Inc., Scarborough, ME, USA) for histidine-rich protein 2 antigen of *P. falciparum* and aldolase enzyme common to all *Plasmodium* spp. Blood smears were stained by a standard technique with Giemsa solution for 30 min and were reviewed by an expert microbiologist. Parasite count was measured by determining the proportion of parasitized erythrocytes or the number of trophozoites per microliter.

### Isolation of Parasite DNA and Molecular Diagnosis Confirmation

DNA isolation from whole blood was performed by using the QIAamp DNA Blood Mini Kit (QIAGEN, Hilden, Germany), according to the manufacturer’s protocol. *P. ovale* molecular diagnosis was confirmed by using seminested multiplex malaria PCR (*18*), which enables the discernment of the 4 most prevalent human malaria species by amplified fragments of DNA in 2 sequential PCRs.

### *P. ovale* Subtype Characterization and Confirmation

Partial sequencing of the ssrRNA gene was used to differentiate *P. ovale curtisi* from *P. ovale wallikeri*. ssrRNA amplification was performed by using a nested PCR specific for *Plasmodium*. The first reaction included UNR (5′-GACGGT ATCTGATCGTCTTC-3′) and PLF (5′- AGTGTGTATCCAATCGAGTTTC-3′) primers, which correspond to the first reaction of the seminested multiplex malaria PCR. The second reaction incorporated the products of the first reaction, along with NewPLFsh (5′-CTATCAGCTTTTGATGTTAG-3′) and NewRevsh (5′-CCTTAACTTTCGTTCTTG-3′) primers. Infection with different malaria species yielded products of 710–740 bp.

The PCR mixture in both reactions consisted of 75 mmol/L Tris-HCl (pH 9.0), 2 mmol/L MgCl_2_, 50 mmol/L KCl, 20 mmol/L (NH_4_)_2_SO_4_, 200 μmol/L dNTP, 0.075 μmol/L of the corresponding PCR primers, 1.25 units *Taq* DNA polymerase (Biotools B&M Labs, S.A., Madrid, Spain), and the template DNA in a reaction volume of 50 μL. The amount of template was 5 μL of DNA extracted by using a QIAamp DNA Blood Mini Kit (QIAGEN). For the second reaction mixture, 2 μL of the PCR product of the first reaction was used as template. For both reactions, a GeneAmp PCR System 2700 thermal cycler (Applied Biosystems, Foster City, CA, USA) was used, beginning with 7 min at 94°C, followed by 40 cycles of 20 s at 94°C, 20 s at 62°C, and 30 s at 72°C for the first round; or 35 cycles of 20 s at 94°C, 20 s at 53°C, and 20 s at 72°C for the second round. The final cycle was followed by an extension time of 10 min at 72°C.

The amplified products were purified by using Illustra DNA and Gel Band Purification Kit (GE Healthcare, Buckinghamshire, UK) and sequenced by using the Big Dye Terminator v3.1 Cycle Sequencing Kit and ABI PRISM 3700 DNA Analyzer (Applied Biosystems). All amplified products were sequenced in both directions twice. To confirm *P. ovale* subtyping, a nested PCR amplification plus sequencing targeting cytochrome (Cyt) b was performed (*3*) in 3 samples of each group, by using a unique second amplification (nested) reaction with primers Cyt b 2F and Cyt b 2R.

### Data Collection

Hospitals that submitted PCR-confirmed and *P. ovale* subtype–identified samples were invited to collaborate in the study. A database was designed and completed after the retrospective review of medical reports and laboratory registries. Patient data collected included sex, age, ethnicity, underlying diseases, time living in non–malaria-endemic countries, dates and purpose of travel, countries visited, malaria chemoprophylaxis, date of admission and diagnosis, symptoms and clinical signs, physical examinations, and complications of severe malaria according to World Health Organization criteria (*19*). The closest possible date of infection was defined as the day of departure from a malaria-endemic area. The time between date of arrival in Spain and onset of illness or diagnosis was calculated once asymptomatic patients were excluded. Patients were classified as early immigrant if they had stayed in a country without malaria for <1 year before diagnosis and as visiting friends and relatives if they had traveled to a malaria-endemic country after 1 year living in a non–malaria-endemic area. Recent *Plasmodium* infection was defined as probable or definite malaria infection in the 3 months before *P. ovale* infection was diagnosed.

Recorded laboratory results included microbiological data; complete blood count with leukocytes, hemoglobin, and platelet levels; levels ​​of creatinine, albumin, transaminases, lactate dehydrogenase, and direct bilirubin in plasma; and glucose-6-phosphate dehydrogenase activity in erythrocytes. Abdominal ultrasonography and serologic studies to detect infection with HIV and hepatitis A, B, and C viruses were reviewed. Recorded treatments and compliance, clinical and microbiological evolution, and duration of hospital stay for those admitted were included.

### Statistical Analysis

Differences of proportions were evaluated by χ^2^ test or Fisher exact test, as appropriate for sample size. Means between groups were calculated by using the Student *t*-test for independent samples if the normal distribution could be assumed; we used the Levene test for homogeneity of variances. If normality was not valid, we used the Mann-Whitney U test for nonparametric variables.

To test for normality, we used either the Shapiro-Wilks test for small samples or the Kolmogorov-Smirnov test with the Lilliefors correction for large samples. Values were reported as means and SDs or, for nonparametric distributions, medians and interquartile ranges (IQRs). A 2-sided p value of <0.05 was considered to indicate statistical significance. Statistical analyses were performed by using SPSS version 15 (SPSS Inc., Chicago, IL, USA).

## Results

During June 2005–December 2011, a total of 102 samples positive by PCR for *P. ovale* were analyzed at the reference laboratory; of these, we were able to amplify and genotype 55 samples. Poor quality of long-term stored DNA prevented amplification of the other samples. Genetic analyses of the *cytb* gene identified *P. ovale curtisi* in 31 samples from 28 patients and *P. ovale wallikeri* in 24 samples from to 22 patients. 

Twelve hospitals agreed to participate in the study and provided complete epidemiologic, microbiological, biochemical, clinical, and therapeutic information for 35 of the 50 patients for which *P. ovale* genetic characterization was available. Of these, 21 patients had *P. ovale curtisi* infection and 14 had *P. ovale wallikeri* infection. Table 1 shows the demographic and epidemiologic data for these 35 patients. Patient age and sex were virtually the same for patients with *P. ovale curtisi* and *P. ovale wallikeri* infections and corresponded mostly to young persons from Africa who traveled to visit their friends and relatives for a long period or immigrants who had recently arrived. 

**Table 1:** Demographic and epidemiologic characteristics of patients with imported *Plasmodium ovale curtisi* or *P. ovale wallikeri* infections, Spain, 2005–2011*

Characteristic	*P. ovale curtisi*, n = 21	*P. ovale wallikeri*, n = 14	p value
Patient sex			0.332
M	10 (47.6)	9 (64.3)	
F	11 (52.4)	5 (35.7 )	
Patient age, y, median (IQR)	36.50 (23.04–52.66)	38.33 (11.79–45.27)	0.377
Age <15	3 (14.3)	4 (28.6)	0.401
Ethnicity			0.721
Black	15 (71.4)	9 (64.3)	
White	6 (28.6)	5 (35.7)	
Type of patient			0.260
Early immigrant	6 (28.6)	4 (28.6)	
Traveler	14 (66.7)	10 (71.4)	
Reason for travel			
Visiting friends and relatives	9 (42.8)	7 (50.0)	
Tourism		1 (7.1)	
Work	3 (14.3)	2 (14.3)	
Cooperation	2 (9.5)		
Unknown	1 (4.8)		
Duration of travel, d, median (IQR)	75 (23.25–91.50)	23 (15.00–81.50)	0.279
Country of infection			0.486
Equatorial Guinea	12 (57.1)	7 (50.0)	
Nigeria	2 (9.5)	3 (21.4)	
Equatorial Guinea or Cameroon	1 (4.8)	0	
Ghana	1 (4.8)	1 (7.1)	
Ethiopia	1 (4.8)	0	
Guinea-Conakry	1 (4.8)	0	
Liberia	1 (4.8)	0	
Angola	1 (4.8)	0	
Guinea-Bissau	1 (4.8)	0	
Guinea-Conakry or Senegal	0	1 (7.1)	
Côte d’Ivoire	0	1 (7.1)	
Mozambique	0	1 (7.1)	
Chemoprophylaxis			0.627
No prophylaxis	17 (81.0)	13 (92.9)	
Mefloquine, incomplete	1 (4.8)	1 (7.1)	
Mefloquine	1 (4.8)	0	
Doxycycline	1 (4.8)	0	
Atovaquone/proguanil	1 (4.8)	0	
Days from arrival to onset of symptoms, median (IQR)	94.5 (12.5–297.2)	9.5 (2.7–58.2)	0.077
Days from onset of symptoms to diagnosis, median (IQR)	8 (2.7–16.5)	3.5 (2.0–7.7)	0.206
Recent *Plasmodium* infection	3 (14.3)	3 (21.4)	>0.999
Other infections			
Hepatitis B virus			>0.999
Active	1/11 (9.1)	0/10	
Cured or vaccinated	6/11 (54.5)	5/10 (50.0)	
Negative	4/11 (36.4)	5/10 (50.0)	
Hepatitis C virus	1/7 (14.3)	0/10	0.412
HIV	1/7 (14.3)	0/10	0.412
Filariasis†	3/6 (50.0)	0/4	0.200
Intestinal parasites‡	3/6 (50.0)	1/4 (25.0)	0.571
Other underlying conditions	9 (42.8)	6 (42.8)	>0.999
Diabetes mellitus	2 (9.5)	1 (7.1)	
Drepanocytosis	2 (9.5)	0	
Hypertension	4 (19.0)	2 (14.3)	
Obesity	1 (4.8)	0	
Acute pancreatitis	0	1 (7.1)	
Policystosis and nephrectomy	0	1 (7.1)	
Oligoarthritis	0	1 (7.1)	
Glucose-6-phosphate dehydrogenase deficiency	2/14 (14.3)	0/8	0.515
Pregnancy	1 (4.8)	0	>0.999

*Values are no. (%) patients or no. positive/total no. (%) patients unless otherwise indicated. IQR, interquartile range. †*Mansonella perstans* (n = 2), *Loa loa* (n = 1). ‡*Trichiuris trichiura* (n = 3), hookworms (n = 2), *Ascaris lumbricoides* (n = 2), *Strongyloides stercoralis* (n = 1), *Entamoeba hystolitica* (n = 1).

The lapses between time of arrival in Spain, onset of illness, and diagnosis were longer for patients with *P. ovale curtisi* infection than for those with *P. ovale wallikeri* infection, but not significantly. Most travelers did not take any malaria prophylaxis or did not adhere to the full regimen. All but 2 infections were acquired in West Africa, and both *Plasmodium s*pecies were found in patients from Nigeria, Equatorial Guinea, Ghana, and Guinea-Conakry. A high rate of underlying disease was found among patients in both parasite groups; the *P. ovale curtisi* group included 3 early immigrants who were chronically infected with HIV, hepatitis B virus, and hepatitis C virus and carried filarial and intestinal parasites. 

Microbiological and laboratory data for the patients are shown in Table 2. Statistically significant worse levels of thrombocytopenia were found among patients with *P. ovale wallikeri* infection compared with those who had *P. ovale curtisi* infection, but no other significant difference was found. One mixed infection with *P. falciparum* was found among each patient group. The RDT technique used showed a low sensitivity (<30%) for detecting *P. ovale* once mixed infections were excluded.

**Table 2:** Microbiological characteristics of patients with imported *Plasmodium ovale curtisi* or *P. ovale wallikeri* infections, Spain, 2005–2011*

Characteristic	*P. ovale curtisi*, n = 21	*P. ovale wallikeri*, n = 14	p value
Positive thick smear, no. (%) patients	16 (76.2)	10 (71.4)	>0.999
Positive by PCR only, no. (%) patients	5 (23.8)	4 (28.6)	>0.999
Parasitemia, μL	2,800 (773.25–5,484.25)	1,243.50 (337.75–6,200.00)	0.699
Mixed infection, no. (%) patients	1† (4.8)	1† (7.1)	>0.999
Rapid diagnostic test result, no. positive/total no. patients (%)			
Common antigen positive	4/16 (25.0)	4/12 (33.3)	0.691
*P. falciparum* antigen positive	1/15 (6.7)	2/12 (16.6)	0.569
Leukocyte count, × 10^9^ cells/L	7.2 (4.9–8.7)	5.5 (4.2–8.2)	0.309
Hemoglobin, g/dL	11.6 (9.7–13.6)	10.9 (9.6–12.1)	0.364
Platelet count, × 10^9^ cells/L	126 (106.0–182.5)	91.5 (54.7–117.7)	**0.031**
Albumin, g/dL	3.7 (3.3–4.1)	3.4 (2.8–3.7)	0.063
Creatinine, mg/dL	0.88 (0.6–1.1)	0.97 (0.5–1.1)	0.730
Lactate dehydrogenase, IU/L	434.5 (358.7–807.7)	563 (462.5–731.7)	0.200
Aspartate aminotransferase, IU/L	24.5‡ (20.0–40.2)	31 (22–41)	0.624
Alanine aminotransferase, IU/L	25.5‡ (16.0–49.7)	23 (18.5–47.0)	0.785
Total bilirubin level, mg/dL	0.68‡ (0.6–1.2)	0.87 (0.6–1.4)	0.426

*Values are median (interquartile range) unless otherwise indicated. Boldface indicates significance. †*P. falciparum* was second infection for both patients. ‡One patient had active hepatitis B virus infection.

Clinical and therapeutic data for the patients are shown in Table 3. All 3 asymptomatic patients had *P. ovale curtisi* infection; 1 infection was detected as an incidental finding in a blood smear for sickle cell disease, and the other 2 were found during studies of anemia after negative results were found by thick film examination and RDT results. The remaining 7 patients with negative thick smear and positive PCR results reported at least fever. Symptomatic patients showed no difference in clinical signs and symptoms, but those with *P. ovale wallikeri* did have a higher mean number of symptoms (3.5 per patient) than did those with *P. ovale curtisi* (2.7). The number of complications was similar in both groups; 3 cases of severe anemia occurred, 2 of them related to sickle cell disease, and 1 case of ADRS occurred in a patient with *P. ovale wallikeri* infection. 

**Table 3:** Clinical and therapeutic characteristics of patients with imported *Plasmodium ovale curtisi* or *P. ovale wallikeri* infections, Spain, 2005–2011*

Characteristic	*P. ovale curtisi*, n = 21	*P. ovale wallikeri*, n = 14	p value
Asymptomatic	3 (14.3)	0	0.259
Fever	18 (85.7)	14 (100.0)	0.259
Tertian fever	1 (4.8)	3 (21.4)	0.279
Maximum temperature, ºC, median (IQR)	38.4 (37.5–40.0)	39.7 (38.9–40.5)	0.088
Chills	3 (14.3)	3 (21.4)	0.664
Sweating	0	1 (7.1)	0.400
Headache	6 (28.6)	4 (28.6)	>0.999
Nauseas	0	3 (21.4)	0.056
Vomitus	0	3 (21.4)	0.056
Astenia	2 (9.5)	3 (21.4)	0.369
Epigastralgia	2 (9.5)	0	0.506
Arthralgia	5 (23.8)	3 (21.4)	>0.999
Myalgia	6 (28.6)	4 (28.6)	>0.999
Diarrhea	1 (4.8)	1 (7.1)	>0.999
Chest pain	1 (4.8)	1 (7.1)	>0.999
Cough	4 (19.0)	3 (21.4)	>0.999
Dyspnea	0	1 (7.1)	0.400
Dizziness	2 (9.5)	0	>0.999
Splenomegaly	5 (23.8)	3 (21.4)	>0.999
Complications or severe malaria	2 (9.5)	2 (14.3)	>0.999
Hemolytic crisis	1 (4.8)	0	
Severe anemia, hemoglobin <7 g/dL	1 (4.8)	1 (7.1)	
Acute respiratory distress syndrome	0	1 (7.1)	
Admission to hospital	13 (61.9)	13 (92.9)	0.056
Duration of hospitalization, d, median (IQR)	4 (3.0–7.5)	5 (3.5–7.5)	0.390
Treatment			0.563
Chloroquine	12 (57.1)	7 (50.0)	
Other treatment	8 (38.1)	7 (50.0)	
Quinine + doxycycline	3 (14.3)	4 (28.6)	
Atovaquone/proguanil	3 (14.3)	1 (7.1)	
Quinine + clindamycin + chloroquine/proguanil	1 (4.8)	0	
Quinine + clindamycin + chloroquine	0	1 (7.1)	
Mefloquine	0	1 (7.1)	
Atovaquone/proguanil + chloroquine	1 (4.8)	0	
No treatment	1 (4.8)	0	
Primaquine	14 (66.7)	10 (71.4)	>0.999
Compliance	19/21 (90.5)	13/13 (100.0)†	0.513

*Values are no. (%) patients or no. positive/total no. (%) patients unless otherwise indicated. IQR, interquartile range. †One patient was lost to follow-up.

Most patients were admitted to a hospital and received inpatient treatment. Roughly half the patients in each group received chloroquine alone. Almost all patients showed good tolerance to treatment and favorable clinical evolution. More than a quarter (33.3% of *P. ovale curtisi* and 28.6% of *P. ovale wallikeri*) did not receive primaquine for radical cure, 2 because of glucose-6-phosphate dehydrogenase deficiency. One patient was lost to follow-up and did not receive any treatment or monitoring.

## Discussion

Our comparative study of the epidemiologic and clinical characteristics of patients with *P. ovale curtisi* and *P. ovale wallikeri* infection found only 1 statistically significant result, a higher rate of severe thrombocytopenia among patients with *P. ovale wallikeri* infection (p = 0.031). Nevertheless, we noted nonsignificant results, including a shorter time from arrival to onset of symptoms in travelers who acquired *P. ovale wallikeri* infection (p = 0.077). This finding fits with findings from a recently published larger series from the United Kingdom in which a significantly shorter latency was found for *P. ovale wallikeri* compared with *P. ovale curtisi* infection (*20*). We also found a trend toward a shorter stay in Africa and shorter interval between onset of symptoms and diagnosis among patients with *P. ovale wallikeri* infection, which could reflect easier transmission, shorter latency, or higher relapse rates. This finding might also mean that slightly more severe illness, including higher median fever (39.7°C vs. 38.4°C) and a greater number of symptoms (3.5 vs. 2.7 per symptomatic patient), led more patients with *P. ovale wallikeri* infection than those with *P. ovale curtisi* infection to seek medical attention earlier. However, the higher percentage of travelers who took at least partial prophylaxis among the *P. ovale curtisi* group (19.2%) might also explain a longer time of onset. A high frequency of nausea and vomiting was found among those with *P. ovale wallikeri* infection, a symptom that was absent among those with *P. ovale curtisi* infection. However, because most cases were asymptomatic or had mild to moderate clinical features, finding significant differences within this narrow clinical spectrum may be especially difficult and might require a much larger study. 

Because the time of infection is more likely to be accurately known, patients with imported malaria might make a better group for study of the epidemiologic and clinical characteristics of different *Plasmodium* species than those living in malaria-endemic countries. Moreover, signs or symptoms may be less affected by other tropical infections, including mixed *Plasmodium* infections, or by patient immunity; imported malaria occurs among a larger number of nonimmune patients and patients who are visiting friends and relatives. Differentiation among primary infection or relapse continues to be practically impossible, but the longer the time of latency, the more probable a relapse.

Although criteria for admission to each hospital involved in the study were different, *P. ovale wallikeri* patients were admitted more frequently. The number of complications was similar in both groups; 3 cases of severe anemia were reported, 2 related to sickle cell disease, and 1 case of ADRS occurred in a patient infected with *P. ovale wallikeri* (*8*). Sickle cell trait was described as a risk factor for infection with *P. ovale* in Senegal (*21*), and a recent series showed 3 of 16 patients with *P. ovale* infection were homozygous for sickle cell disease (*22*), a clearly disproportionate number.

Regarding symptomatic patients, the Bangladesh study (*5*) showed a trend toward a larger number of asymptomatic *P. ovale curtisi* infections (90% vs. 75% for *P. ovale wallikeri*), which would be consistent with our results. In that study, all 13 mixed infections of *P. ovale* with other *Plasmodium* species were asymptomatic; in our study, the 2 patients who had mixed infections with *P. falciparum* had at least fever. The number of *P. ovale* monoinfections in our study confirmed by PCR is high, unlike the number in malaria-endemic areas, where most infections are mixed (*5*,*7*,*23*) and a high rate of submicroscopic carriage occurs (*24*). In part, our finding may result from previous antimalarial treatment or prophylaxis in a number of patients, which could have minimized the number of *Plasmodium* parasites in the blood. 

Among the laboratory results, the only significant difference was found in platelet count, with more severe thrombocytopenia seen among patients with *P. ovale wallikeri* infection than among those with *P. ovale curtisi* infection. Thrombocytopenia is a common finding in patients with malaria; a previous series found 10 (66.6%) of 15 patients with imported *P. ovale* infection had platelet counts of <140,000/μL (*22*). The mechanisms that produce thrombocytopenia in malaria are not known but seem related to a greater severity of illness (*25*). Some studies also suggest an inverse correlation between the level of parasitemia and platelet count (*26*).

We found that indirect parameters of hemolysis, such as hemoglobin, lactate dehydrogenase, and bilirubin levels, were less impaired among patients with *P. ovale curtisi* infection than those with *P. ovale wallikeri* infection, even including 2 patients with sickle cell disease, who had more severe anemia. Albumin values, diminished by other multiple types of infections, ​​also tend to be lower in patients with *P. ovale wallikeri* infection. For transaminases, if we were to exclude a patient with chronic hepatitis B and hypertransaminasemia, we would also find higher values ​​for patients with *P. ovale wallikeri* infection. These data collectively raise the hypothesis that *P. ovale wallikeri* is slightly more pathogenic, which warrants further investigation.

Parasitemia levels were not significantly different between the 2 groups (p = 0.699). In 2 recent studies of *P. ovale* infection that included determination of parasitemia levels and genetic analysis (*5*,*15*), no differences were described. Case reports of high parasitemia levels in patients with *P. ovale wallikeri* infection in Southeast Asia are isolated and noncomparative (*12*–*14*).

Sooner and better access to health care in industrialized countries might provide a broader range of diagnostic tools for malaria, including RDT, thick and thin film examination, or PCR. Current techniques of RDT still show a low sensitivity for detecting *P. ovale* (*27*,*28*). This problem is usually explained by the genetic variability of the 2 subspecies and the low levels of parasitemia detected. After discarding mixed infections, in our study, the aldolase-based RDT used as a common antigen of *Plasmodium* obtained 20% sensitivity for detection of *P. ovale curtisi* and 27.27% for *P. ovale wallikeri*. Results are poorer than those recently published by Bauffe et al., who found a higher false-negative rate of infection for *P. ovale curtisi* than for *P. ovale wallikeri* (60% vs. 43%, respectively) and no significant differences in parasitemia levels (*15*).

Although circulation of *P. ovale* was known in these countries, our study provides PCR confirmation for *P. ovale curtisi* infections from Guinea-Conakry, Ethiopia, and Liberia and *P. ovale wallikeri* infections from Mozambique. Both species have been described as sympatric in time and space in Equatorial Guinea, Republic of Congo, Uganda, Bangladesh, and Angola (*5*,*11**,**29*), maintaining genetic differentiation, which supports the hypothesis of 2 distinct subspecies. Moreover, 2 cases of *P. ovale curtisi* and *P. ovale wallikeri* co-infection have been reported (*5*,*29*).

We found a high rate of underlying chronic diseases among the patients in this study, especially homozygous sickle cell anemia and diabetes mellitus. Previous studies have shown that carriage of the sickle cell trait confers increased susceptibility to *P. ovale* infection (*20*) and that diabetes and HIV infection confer increased susceptibility to *P.* *falciparum* infection (*30*). 

In industrialized countries, improved clinical and microbiological control after treatment and radical cure with primaquine can be achieved for malaria. *P. ovale* seems to remain sensitivity to chloroquine and other antimalarial drugs. Patients in our study showed good clinical response that could be followed up without any relapse, including among those who had complications. The treatment differences reflect the different hospital managing protocols, patient age and pregnancy status, or difficulty in identifying *P. ovale* initially.

Our study has limitations. First, the small number of patients may lack sufficient statistical power to show differences between infections with different *Plasmodium* species. Second, the low performance of genetic amplification may have caused some sample selection bias (e.g., a higher number of samples that were stored short term or came from patients with higher parasitemia levels). Third, patient selection was not systematically planned but was done on the basis of decisions of physicians from many hospitals who sent samples to the reference laboratory and subsequently agreed to participate in this study. Fourth, the study’s retrospective design led to gaps in the information collected. Fifth, only strains of *P. ovale* from Africa were analyzed, and patients were from Africa and Europe; a study of infections and patients from Asia or Oceania might show different results. Last, more diversity in the geographic origin of the strains and a mix of nonimmune and semiimmune patients would lead to more heterogeneous study groups.

In summary, after comparing epidemiologic, clinical, and analytic data for patients with *P. ovale wallikeri* and *P. ovale curtisi* infections, we found significantly more marked thrombocytopenia among patients with *P. ovale wallikeri* infection, but we found no other significant differences. However, some trends toward slightly greater pathogenicity were observed for *P. ovale wallikeri* infection. The description of both genotypes occurring in sympatry without hybrid forms in an increasing number of countries supports the idea of ​​2 well-defined species. Larger prospective studies should be conducted to more fully explore this hypothesis.
